# Prevalence of Viral Hepatitis in Unselected, Consecutively Enrolled Patients Hospitalised for SARS-CoV-2

**DOI:** 10.1007/s10900-022-01111-6

**Published:** 2022-06-21

**Authors:** Silvia Dettori, Chiara Russo, Sara Mora, Mauro Giacomini, Lucia Taramasso, Chiara Dentone, Antonio Vena, Matteo Bassetti, Antonio Di Biagio

**Affiliations:** 1 Department of Health Sciences (DISSAL), University of Genoa, Genoa, Italy; 2 Infectious Diseases Unit, Ospedale Policlinico San Martino IRCCS, Genoa, Italy; 3 Department of Informatics Bioengineering, Robotics, and Systems Engineering (DIBRIS), University of Genoa, Genoa, Italy; 4 Division of Infectious Diseases, Department of Health Sciences (DISSAL), University of Genoa, Genoa, Italy; 5 IRCCS Ospedale Policlinico San Martino, Largo Rosanna Benzi, 10, 16132 Genoa, Italy

**Keywords:** Viral hepatitis, Hepatitis screening, SARS-CoV-2, Eradication

## Abstract

Diagnosing people living with chronic viral hepatitis is challenging due to the absence of symptoms as long as liver decompensated cirrhosis come out. The aim of this retrospective study was to evaluate the prevalence of HBV and/or HCV infections in a non-selected population, hospitalised for SARS-CoV-2 infection in a tertiary care hospital in Northern Italy. During the study period 1,429 patients were admitted to hospital for SARS-CoV-2 infection, serologic tests for HBV and/or HCV were available for 382 (27%) patients and 3 were excluded due to their previous known serologic status. Among 379 patients, 235 (62%) were male, median age was 70 years (range 21–103), 360 (95%) were Caucasian. Among them, 372/379 (98%) were screened for HBsAg, 320/379 (84%) for HBcAb. HBsAg was positive in 2/372 (0.5%, 95% CI 0.0006–0.02) patients (only in one HBV-DNA was performed that was negative), while HBcAb was found positive in 55/320 (17%, 95% CI 0.13–0.22). Among 370/379 (98%) patients screened for HCV, 11/370 (3%, 95% CI 0.02–0.05) had positive HCV-Ab. Five out of 11 (45%) were tested for HCV-RNA that resulted positive in two patients (0.5%, 95% CI 0.0006–0.02). Considering this data, even though the screening was performed in only 27% of study population, a tailored screening in people with known risk factors for hepatitis might be preferable to universal screening in low prevalence areas. Also a prompt diagnostic workout should begin in case of clinical or laboratory suspicion of hepatitis and in those starting immunosuppressive treatments.

## Introduction

Hepatitis B (HBV) and C (HCV) virus infections remain a significant cause of morbidity and mortality in the world [1]. The World Health Organization (WHO) estimates that in 2019, 1,500,000 new HBV infections and 1,500,000 new HCV infections occurred, with 820,000 and 290,000 HBV and HCV related deaths, respectively [1]. In the United States alone, the Center for Disease Control and Prevention (CDC) estimates 20,700 acute HBV infections and 57,500 acute HCV infections in 2019, with an incidence of 1.0 and 1.3 cases/100,000 people, respectively [2]. According to the CDC, the most affected groups are people aged 40–49 years, males, white non-Hispanics and injecting drug users (IDU) [2]. The Polaris Observatory, the authoritative resource for epidemiological data for HBV and HCV, estimates that 71 million people are infected with HCV globally and approximately 400,000 people/year die from causes related to HCV [3].

In Italy, the epidemiology system for surveillance of viral acute hepatitis SEIEVA (coordinated by the Italian Superior Health Institute) reported an incidence of 0.21 and 0.04 cases/100,000 inhabitants for acute HBV and HCV hepatitis in 2020 [4]. These numbers confirm a trend in reduction of HBV and HCV incidence in Italy as compared to the past, as in 1985 the estimated incidence of HBV and HCV was 12 and 5 cases per 100,000 inhabitants [4]. Despite this, the true prevalence of chronic HBV and HCV infections in Italy as well as globally is still to be determined, and only estimations can be made at present. It is estimated that in 2020, 332,157 people were infected with HBV in Italy, of which only 29% have been diagnosed, while only 46% of the total cases have been treated, with 1,200 annual deaths related to HBV [5]. For HCV, it is estimated that 680,000 people were infected in 2020, of which only 60% have been diagnosed and only 5% of the total have been treated, with 11,236 annual deaths related to HCV [5].

In 2016, the WHO set the goal of eradication of viral hepatitis as a major public health objective. The specific goals are 90% of diagnosed infections, 80% of treated infections and 65% reduction of hepatitis-related mortality by 2030 [6].

Unfortunately, only 24% of 45 high-income countries are forecasted to reach the WHO elimination targets by 2020 and 62% by 2050 [7].

Strategies to improve the detection of unknown HBV and HCV infections are currently under investigation. To evaluate the prevalence of HBV and HCV in the general population and the possible utility of serologic screening tests for people accessing hospital services, we studied the seroprevalence of HBV and HCV in a non-selected population of patients hospitalised for Severe Acute Respiratory Syndrome Coronavirus 2 (SARS-CoV-2) infection with Coronavirus disease 2019 (COVID-19) in a tertiary care hospital in Northern Italy.

## Materials and Methods

This is a retrospective, single-centre study conducted in IRCCS San Martino Hospital, a 1200-bed tertiary care hospital in Genoa, Northern Italy. The study period was from 25th February 2020 to 31st January 2021.

All patients hospitalised for SARS-CoV-2 infection were consecutively included in the study if they were aged ≥ 18 years. Exclusion criteria were their known positive serological statuses for HBV or HCV. Data on sex, age, ethnicity, HBsAg and HBcAb, HCV-Ab, HCV-RNA and HBV-DNA were retrieved from MedInfo, an online database for anonymous and automatic data collection [8]. In addition, possible risk factors for HBV and HCV infections were collected, such as IDU or people born between 1945 and 1965, sometimes referred to as baby boomers [9], or exposure to transfusion or injection with glass syringes in the years between the 50s and the 80s [10].

The primary objective was to describe the prevalence of HBV and/or HCV infections in a non-selected population. The secondary objective was to evaluate the number of coinfections with HIV. We also evaluated if newly diagnosed patients were hospitalized and treated, and whether their progress was followed up on. Data are presented as prevalence with a 95% confidence interval (CI) calculated with a binomial test.

The collection of anonymized data for the study was approved by the Ethics Committee of the Coordinating Centre and specific informed consent was waived due to the retrospective nature of the study (registry number 163/2020, Liguria Region Ethics Committee).

## Results

During the study period, 1429 patients were admitted to our hospital for SARS-CoV-2 infection. Serologic tests for HBV and/or HCV were available for 382 (27%) patients.

Three patients were excluded due to their known previous HCV positive serological statuses: two of them had been previously treated with Direct Acting Antivirals (DAA) and had concomitant HIV infection on antiretroviral treatment, while the third had a previous negative HCV-RNA status. We reported data about the study population of 379 patients. Among them, 235 (62%) were male, the median age was 70 years (range 21–103), 360 (95%) were Caucasian (357 Italian), 17 (4%) Latin-American and 2 (0.5%) were from Africa and Asia.

### HBV

**Fig. 1:**
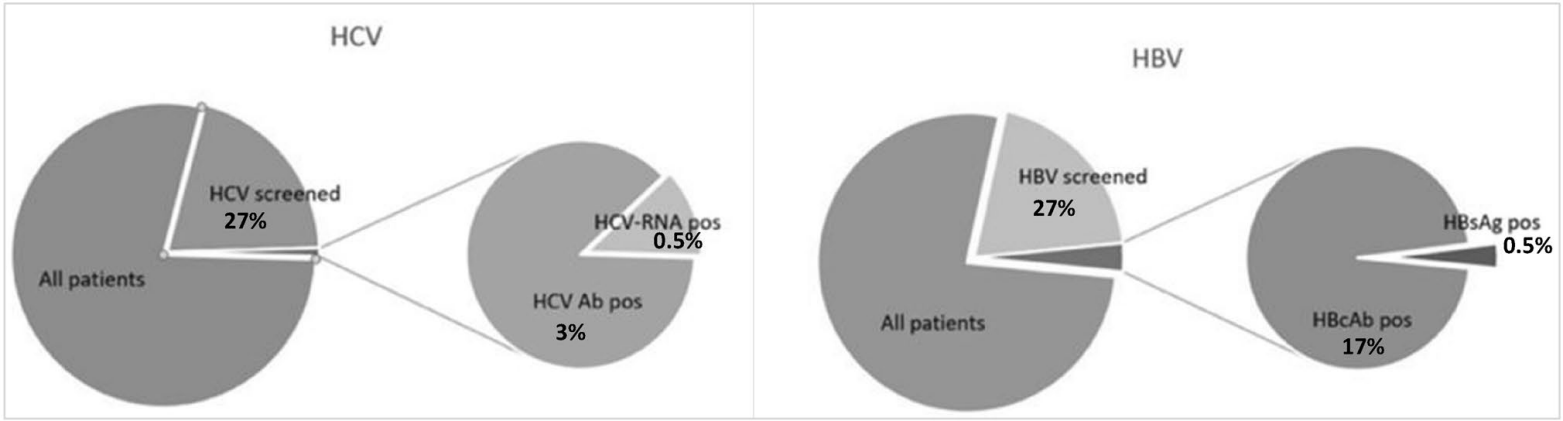
HBV and HCV screening performed and results. *Ab* antibody, *Ag* antigen, *pos* positive

Among patients with whom serologic tests were performed, 372/379 (98%) were screened for HBsAg and 320/379 (84%) for HBcAb. HBsAg was positive in 2/372 (0.5%, 95% CI 0.0006–0.02) patients, while HBcAb was found in 55/320 (17%, 95% CI 0.13–0.22) screened patients (Fig. 1).

Among the two patients with HBsAg, HBV-DNA was negative in one patient and was not tested in the other. Neither of them was on anti-HBV treatment nor had chronic HCV or HIV infection (Table 1), and neither received specialistic follow-ups.

**Table 1:** Patients’ characteristics

Patient	Age	Sex	Ethnicity	Medical history	HCV-RNA/HBV-DNA (UI/mL)	Risk factor for chronic hepatitis	Clinical follow-up/therapy
HCV-Ab positive							
Patient 1	67	F	Caucasian	Ex smoker, previous appendicectomy	HCV-RNA 7.4 × 10^6^	Age*	Lost to follow up
Patient 2	60	M	Caucasian	Heart failure, atrial fibrillation, T2DM	HCV-RNA 6.4 × 10^5^	Age*	HCV positive serology since 2015, unknown to patient. Scheduled ambulatorial follow-up, death for other cause before data of visit
Patient 3	71	M	Caucasian	Epilepsy, poliomyelitis, post-traumatic cerebral bleeding	Not done	Age*	Death the day after serology performed
Patient 4	103	F	Caucasian	T2DM, psoriasis	Not done	Age*	HCV positive serology since 2015, unknown to patient
Patient 5	85	F	Caucasian	Myocardial ischemia, CKD	Not done	Age*	Death 4 days after serology performed
Patient 6	54	M	Caucasian	Asthma, ex-IDU	Negative	IDU	None required
Patient 7	86	M	Caucasian	Atrial fibrillation, COPD, CKD	Negative	Age*	None required
Patient 8	80	F	Caucasian	COPD, T2DM, obesity	Not done	Age*	Death during a second hospitalization in June 2021
Patient 9	84	M	Caucasian	Atrial fibrillation, heart failure, T2DM	Not done	Age*	Death during hospitalization
Patient 10	82	M	Caucasian	COPD, T2DM, alcohol abuse	Negative	Age*	HCV positive serology since 2014, unknown to patient
Patient 11	21	F	Caucasian	IDU, bipolar disorder	Not done	IDU	Lost to follow up
HBsAg positive							
Patient 1	60	M	Caucasian	Hypertension, Elher-Danlos syndrome and mitral prolapse, spinocellular carcinoma with lymph nodal metastasis	Not done	Age*	Death during hospitalization
Patient 2	49	M	Caucasian	Psoriasis	Negative	Unknown	None

*Ab* antibody, *Ag* antigen, *CKD* chronic kidney disease, *COPD* chronic obstructive pulmonary disease, *DAA* direct acting antivirals, *F* female, *IDU* intravenous drug users, *M* male, *T2DM* type 2 diabetes mellitus

*Age: people born between 1945 and 1965 or with possible exposure to transfusion or injection with glass syringes between 50s’ and 80s’

### HCV

Among patients with whom serologic tests were performed, 370/379 (98%) were screened for HCV and 11/370 (3%, 95% CI 0.02–0.05) had positive HCV-Ab. Five out of 11 (45%) patients with HCV-Ab positivity were tested for HCV-RNA (Fig. 1). HCV-RNA resulted positive in two patients out of 370 (0.5%, 95% CI 0.0006–0.02) leading to diagnoses of HCV chronic infections, but neither of them began anti-HCV treatment at the time of the study. Patients’ characteristics are described in Table 1.

### CO-INFECTIONS

Among patients with positive HCV-Ab, 5/11 (45%) also had a previous HBV infection, with positive HBcAb and negative HBsAg, but none had concomitant HIV infection. Four of them were aged > 60 years and one was a previous IDU.

## Discussion

Our study highlights a low prevalence of HBV and HCV in the study population (0.5% and 0.5%, 95% CI 0.0006–0.02, respectively).

We do not have data about HCV screening in the pre-COVID-19 era from our hospital, but we can assume that the frequency of screening during the study period was reduced compared to the past [11]. Conversely, in other areas of Northern Italy, the SARS-CoV-2 pandemic was thought to be exploited to increase HCV screening in the Italian population and achieve the WHO goal of HCV elimination [12]. Among patients with a positive HCV serology in our study population, five out of 11 showed severe clinical conditions, thus further investigations were not conducted. On the other hand, almost all HCV-Ab positive patients had anamnestic reports of medical visits or hospitalisations before the study was performed, and therefore had missed the opportunity for a previous diagnosis and eradication.

While analysing the patients’ characteristics, we found that more than half of HCV-Ab positive patients were aged > 60 years, according to the high prevalence of HCV in the second part of the twentieth century [2, 10], and about 20% were IDU, a known risk factor for HCV infection [2, 10].

Although the prevalence of chronic HBV and HCV is still unknown, some authors hypothesised that there is a high number of HCV infected individuals still undiagnosed in Italy [10]. Through a mathematical model, they estimated undiagnosed HCV to be about 281,809 individuals in October 2019 [10].

Even if extensive screening can reduce hospitalisation, costs and deaths related to chronic hepatitis [13], the perception based on our results is that the screening could be narrowed down to suit people with HCV infection risk factors, such as period of birth, drug abuse, blood transfusion and/or surgical procedure before the 90s [2, 10, 14]. In addition, it is important that patients with a new diagnosis of chronic HCV infection are referred to care [15].

Furthermore, the prevalence of chronic HBV infection was low in our study since only two patients had positive HBsAg, and we expected an even lower prevalence in people born in Italy after HBV vaccination became mandatory in 1990 [16].

Similar to HCV chronic hepatitis, we can assume that the screening for HBV was higher in the pre-pandemic period [17]. Moreover, a recent study conducted in the United States demonstrates that conducting HBV screenings for a population is cost-effective even when the estimated prevalence is low, because it can help to prevent chronic HBV infection complications [18].

Even when the prevalence of active chronic HBV infection was low, we found a high proportion of previous HBV infections with HBcAb positivity (about 20%). People with HBcAb positivity are at risk of reactivation of HBV infection when exposed to immunosuppressive therapies [19, 20]. As some immunosuppressive drugs used for COVID-19, like tocilizumab and baricitinib can lead to HBV reactivation [21], HBV screening should be offered with significant attention paid to patients eligible for these therapies, even though the risk appears to be low in patients with previous and resolved HBV infections [22]. Most data stems from studies about autoimmune diseases in which these medications were administered together with some other agents, such as high dose and prolonged corticosteroids therapy or other disease-modifying antirheumatic drugs (DMARDs) [23, 24]. In our study population, only 320/1429 (22%) patients potentially to be treated with immunosuppressive therapies were screened for HBcAb, a low percentage which was probably due to the pandemic era. However, even in an overworked period, clinicians should maintain diagnostic and therapeutic appropriateness. Nonetheless, not all patients with SARS-CoV-2 infections need immunosuppressive therapies, especially after the introduction of vaccination and outpatients’ therapies [25].

The first limitation of our study is that serology for HBV and/or HCV was tested in only 27% of patients admitted to the hospital for SARS CoV-2 infection in the study period. Furthermore, our study is retrospective and the seroprevalence screening of HBV and HCV chronic infection in the pre-pandemic era is unknown to us. The second limitation is that during the first wave of the COVID-19 pandemic, medical efforts were focused on COVID-19 instead of routine patient management and the best clinical approach. This could have also reduced the linkage of patients with a new diagnosis of chronic HBV and HCV hepatitis to care [26]. Yet another limitation is that 95% of patients were Caucasian, and only 0.5% were from Africa and Asia, a population with a higher prevalence of chronic HBV and HCV infection compared to Italy [27, 28].

Since the median age of our study population is 70 years, we conducted the screening on the population with the higher prevalence of chronic HBV and HCV infection in Italy (9, 10), and therefore we can assume that the prevalence of chronic viral hepatitis is low among the non-selected population.

In conclusion, diagnosing people living with chronic viral hepatitis is challenging due to the absence of symptoms other than liver decompensated cirrhosis. A tailored screening in people with known risk factors for hepatitis might be preferable to universal screening in low prevalence areas, even though prompt diagnostic workout should begin in case of clinical or laboratory suspicion of hepatitis and in those patients starting immunosuppressive treatments. Moreover, a targeted effort could give benefits on the linkage to care and epidemiological control and reduce the risk of losing patients to follow-up in a wide screening.

Nevertheless, our study reports the prevalence and not the incidence of HBV and HCV chronic hepatitis. Even if we think that a tailored screening should be preferred instead of a universal one, surveillance of chronic hepatitis should be performed to identify a possible trend reversal.

## Data Availability

Anonymized data available to all.

## Code Availability

Individual code available to all.
